# Co-relation between periodontitis and maxillary sinusitis: A cross sectional CBCT based study

**DOI:** 10.6026/973206300222880

**Published:** 2026-05-31

**Authors:** Madhu Varma, Dhalkari C.D, Maya S Indurkar, Aishwarya R Mulay, Pawan Kumar Chande

**Affiliations:** 1 Department of periodontology Government Dental College and Hospital, Chhatrapati Sambhajinagar, Maharashtra, India

**Keywords:** Mucosal thickness, periodontitis, maxillary sinusitis, cone-beam computer tomography (CBCT), alveolar bone loss

## Abstract

Periodontitis is a potential but often underdiagnosed cause of maxillary sinusitis, complicating accurate diagnosis due to overlapping clinical features. Therefore, it is of interest to evaluate the correlation between alveolar bone loss and maxillary sinus mucosal thickening in 50 patients with periodontitis. Measurements were performed in coronal and sagittal sections to assess the extent of periodontal destruction and sinus involvement. A significant association was observed, with greater mucosal thickening in patients exhibiting moderate to severe alveolar bone loss compared to mild cases. Thus, we show the importance of through periodontal evaluation in patients presenting with maxillary sinusitis to ensure accurate diagnosis and management.

## Background:

Periodontal diseases represent a significant global health burden, affecting approximately 20% to 50% of the population across both industrialized and developing nations, impacting individuals across all age groups from adolescence through elderly [1]. Characterized by the pathologic loss of the periodontal ligament and alveolar bone, periodontitis stands as one of the most prevalent chronic inflammatory conditions worldwide [2]. While the oral health implications of periodontal disease are well-established, emerging evidence suggests that its impact extends beyond the oral cavity, particularly concerning the maxillary sinus. Maxillary sinusitis, defined as symptomatic inflammation of the maxillary sinus lasting longer than 12 weeks in its chronic form, has traditionally been considered primarily rhinogenous in origin [4]. However, accumulating evidence indicates that dental infections serve as a major predisposing factor in a substantial proportion of cases. Recent epidemiological data reveals that dental issues underlie nearly one-third of unilateral maxillary sinusitis cases, with odontogenic sinusitis (OS) accounting for 10%-12% of all sinusitis presentations [3, 5]. More striking are recent findings suggesting that the prevalence of OS may be as high as 41%, indicating a potentially underestimated clinical problem [5]. Both periodontitis and odontogenic sinusitis share important pathophysiological characteristics, being multifactorial and polymicrobial in nature [6, 7, 8, 9, 10]. The anatomical proximity of maxillary posterior teeth to the maxillary sinus, coupled with shared vascular supply, provides a direct pathway for inflammatory transmission from periodontal pockets to the sinus cavity [11, 12, 13]. This intimate anatomical relationship explains why teeth with roots extending into or near the maxillary sinus demonstrate approximately twice the likelihood of association with maxillary sinus pathologies. Indeed, apical periodontitis and other periodontal disorders account for an estimated 83% of OS cases, underscoring the critical importance of recognizing this connection [10]. Despite its significant prevalence, odontogenic sinusitis remains underappreciated in current medical and dental literature [8]. The diagnostic challenge is compounded by the similarity of symptoms between odontogenic and non-odontogenic sinusitis, substantially increasing the risk of misdiagnosis and inappropriate management [9]. Several modifiable risk factors, including smoking, diabetes mellitus and poor oral hygiene, contribute significantly to periodontitis development [2]. Therefore, it is of interest to report the relationship between periodontitis and maxillary sinus health in an unexplored population.

## Materials and Methods:

## Ethical approval:

The present research was done in compliance with the Scientific Research Ethics standards of the Institutional Ethical Committee. During data collection, analysis and publication of findings, all the required actions were taken to preserve patient privacy and confidentiality.

## Study design and setting:

This was a cross-sectional study carried out at Department of Periodontology, Government Dental College and Hospital, Chhatrapati Sambhajinagar. The CBCT data were collected from Digital Imaging and Communications in Medicine (DICOM) archive folder of Government Dental College and Hospital. The patients were advised CBCT for either the pathologies or for planning the implant surgery.

## Sample selection and population of the study:

The selection of patients was done in Department of Periodontology; those who were diagnosed with periodontitis recruited for the study and were sent for CBCT, the older CBCT data were also recruited from the archive folder of Digital Imaging and Communications in Medicine (DICOM). The diagnosis of all cases of periodontitis was done using the conventional clinical and radiograph parameters, i.e., bone loss assessment on the cone-beam computer tomography (CBCT) images. Diagnostic confirmation was done through thorough examination of the progress notes and clinical documentation of each patient.

## The periodontal bone loss was calculated in terms of percentage of root length and classified as:

[1] Normal to mild, <25% bone loss

[2] Moderate, 25 - 50% bone loss

[3] Severe, >50% bone loss

## Inclusion criteria:

The patients who participated in the study had to fulfil the following requirements:

[1] Clinical and radiographic diagnosis of periodontitis.

[2] Presence of CBCT images showing clearly at least one maxillary sinus and its corresponding poster maxillary teeth.

## Exclusion criteria:

[1] Patients were not included in the study in case any impacted, unerupted teeth in maxillary posterior region.

[2] Entire edentulism of the posterior maxillary (absence of all teeth of the maxilla in the posterior part of the maxilla).

[3] Known systemic diseases associated with metabolism of bones: Rheumatoid arthritis, Osteoporosis Systemic lupus erythematosus. Presence of dental implants in the maxillary posterior. Periapical- Periodontitis or periapical Abscess

## Data collection:

CBCT of patient suffering from periodontitis were evaluated. The percentage of bone loss was evaluated by calculating the percentage of bone loss, by dividing distance from CEJ to alveolar crest with total root length. Based on percentage of Bone loss, it was categorised into Mild (<25%), Moderate (25-50%) and Severe (>50%). For measurement of Mucosal thickening, Coronal and sagittal section of maxillary premolar and molars were evaluated by drawing a line parallel to long of the tooth and the radio-opacity inside the sinus wall was measured.

## Results:

A total of 34 CBCT scans of patients diagnosed with periodontitis were evaluated to assess the correlation between periodontal bone loss and maxillary sinus mucosal thickening. The mean age of the study population was 39.47 ± 8.12 years (Table 1). Periodontal bone loss was categorized into mild (<25%), moderate (25-50%) and severe (> 50%) according to the percentage of root length affected. A very high prevalence of mucosal thickening was observed in the study population. 94.1% of patients showed thickening in the left maxillary sinus. 100% showed thickening in the right maxillary sinus. This strongly suggests a possible association between periodontal bone loss and maxillary sinus mucosal changes. The present CBCT-based cross-sectional study included 34 patients diagnosed with periodontitis. The mean age of the participants was 39.47 ± 8.12 years. Based on the severity of periodontal bone loss, 14.7% of patients exhibited mild bone loss (<25%), while 58.8% showed moderate bone loss (25-50%) and 26.5% demonstrated severe bone loss (>50%) (Table 2). CBCT evaluation of the maxillary sinus revealed that the mean mucosal thickness was 3.90 mm on the left side and 4.37 mm on the right side. Considering the pathological threshold of >2 mm, mucosal thickening was observed in 94.1% of the left maxillary sinuses and in 100% of the right maxillary sinuses. These findings suggest a strong association between periodontal bone loss and maxillary sinus mucosal thickening (Table 3).

## Discussion:

The association between periodontitis and maxillary sinus pathology has increasingly gained attention in contemporary dental research, particularly with the advent of advanced imaging modalities such as cone-beam computed tomography (CBCT). The anatomical proximity of maxillary posterior teeth to the sinus floor establishes a direct pathway through which periodontal disease can influence sinus health. Radiographic studies have consistently demonstrated that roots of premolars and molars often lie in close approximation to, or even protrude into, the maxillary sinus, thereby predisposing the sinus mucosa to inflammatory changes secondary to periodontal destruction [14]. This intimate relationship forms the biological basis for the development of odontogenic sinusitis in patients with periodontal disease. Several CBCT-based investigations have confirmed a significant correlation between periodontal bone loss and maxillary sinus mucosal thickening. Studies evaluating odontogenic conditions using three-dimensional imaging have shown that periodontal inflammation contributes to mucosal alterations, with increased bone loss associated with greater sinus involvement [15]. Systematic reviews and meta-analyses further support this association, highlighting that odontogenic infections, including periodontitis, are among the most common causes of maxillary sinus pathology and may account for a substantial proportion of sinusitis cases [16]. These findings reinforce the concept that periodontal disease should not be considered an isolated oral condition but rather a contributor to adjacent anatomical pathologies. The role of CBCT in diagnosing the perio-antral relationship is particularly significant. Unlike conventional two-dimensional imaging, CBCT allows precise visualization of both hard and soft tissues, enabling accurate assessment of mucosal thickening, sinus floor integrity and periodontal bone loss. Retrospective cross-sectional studies have demonstrated a strong correlation between CBCT findings in the maxillary sinus and underlying dental conditions, underscoring the diagnostic superiority of this modality in identifying odontogenic sources of sinus disease [17]. Furthermore, systematic reviews have emphasized the prevalence of sinus pathologies and anatomical variations detected through CBCT, many of which may remain undiagnosed using traditional radiographic techniques [18]. The pathological progression from periodontitis to sinus involvement appears to follow a continuum. As periodontal disease advances, progressive alveolar bone loss may extend toward the sinus floor, eventually leading to thinning or perforation of the cortical boundary. This structural compromise facilitates the migration of oral microorganisms and inflammatory mediators into the sinus cavity, thereby initiating or exacerbating mucosal inflammation. Large retrospective analyses have demonstrated a higher prevalence of sinus membrane thickening in association with unhealthy teeth, further substantiating this pathogenic pathway [19]. Clinically, this highlights the importance of early periodontal intervention to prevent secondary sinus complications. From a clinical perspective, these findings necessitate a multidisciplinary approach in the management of patients presenting with either periodontitis or maxillary sinusitis. Case series and clinical observations have emphasized that odontogenic sinusitis often remains underdiagnosed due to overlapping symptoms with rhinogenic sinusitis, thereby requiring careful evaluation of dental etiologies in refractory cases [20]. CBCT imaging plays a crucial role in differentiating odontogenic from non-odontogenic sinusitis by localizing pathology around the roots of maxillary teeth and identifying disruptions in the sinus floor. Additional studies have further reinforced the association between odontogenic conditions and sinus disease, demonstrating that mucosal thickening is significantly more prevalent in patients with periodontal or periapical pathology [21, 22]. Moreover, retrospective CBCT analyses have shown a consistent correlation between dental infections and sinus abnormalities, suggesting that periodontal health directly influences sinus physiology [23]. Advances in understanding the microbiology and pathogenesis of odontogenic sinusitis also indicate that polymicrobial infections originating from periodontal pockets can alter the sinus environment, leading to chronic inflammation and impaired drainage [24]. Importantly, CBCT-based evidence has provided direct quantitative support for this relationship. Studies have demonstrated that periodontal bone loss is significantly associated with increased sinus mucosal thickness, with greater severity of periodontal destruction correlating with higher degrees of mucosal involvement [25]. This dose-response relationship not only validates earlier clinical observations but also provides objective radiographic evidence supporting the perio-antral connection. In summary, the relationship between periodontitis and maxillary sinusitis is well-established and multifactorial, involving anatomical proximity, microbial invasion and inflammatory pathways. The integration of CBCT into routine diagnostic protocols has significantly enhanced the ability to detect and understand this association. These findings underscore the necessity for comprehensive periodontal evaluation in patients with sinus pathology and highlight the importance of maintaining periodontal health to preserve both oral and sinus integrity.

## Conclusion:

The correlation between periodontitis and maxillary sinus membrane thickening is discussed. A through clinical and radiographical examination of alveolar bone loss to be conducts before planning for treatment. Further clinical studies are required to evaluate the exact relationship between the mucosal thickness and alveolar bone loss.

## Figures and Tables

**Table 1:** Distribution of study participants according to severity of periodontal bone loss

**Bone Loss Category**	**Number of Patients (n)**	**Percentage (%)**
Mild (<25%)	5	14.7
Moderate (25-50%)	20	58.8
Severe (>50%)	9	26.5
Total	34	100

**Table 2:** Mean maxillary sinus mucosal thickness measured on CBCT

**Sinus Side**	**Mean Thickness (mm)**	**Standard Deviation (mm)**
Left Maxillary Sinus	3.9	1.25
Right Maxillary Sinus	4.37	1.41

**Table 3:** Prevalence of pathological mucosal thickening (>2 mm)

**Sinus Side**	**Patients with Thickening**	**Percentage (%)**
Left Maxillary Sinus	32	94.1
Right Maxillary Sinus	34	100
Total Cases Evaluated	34	100
